# A randomized controlled pilot study of daily intravenous ketamine over three days for treatment-resistant depression

**DOI:** 10.1186/s12888-024-05951-5

**Published:** 2024-07-18

**Authors:** Keerati Pattanaseri, Juthawadee Lortrakul, Kankamol Jaisin, Maytinee Srifuengfung, Naratip Sa-nguanpanich, Natee Viravan, Pornjira Pariwatcharakul, Wattanan Makarasara, Woraphat Ratta-apha

**Affiliations:** 1 Faculty of Medicine Siriraj Hospital, Department of Psychiatry, Mahidol University, Bangkok Noi, Bangkok, Thailand; 2 Faculty of Medicine Siriraj Hospital, Department of Anesthesiology, Mahidol University, Bangkok Noi, Bangkok, Thailand; 3 Faculty of Medicine Siriraj Hospital, Siriraj Informatics and Data Innovation Center, Mahidol University, Bangkok Noi, Bangkok, Thailand; 4 Faculty of Medicine Siriraj Hospital, Research Department, Mahidol University, Bangkok Noi, Bangkok, Thailand

**Keywords:** Depression, Efficacy, Ketamine, Response, Tolerability

## Abstract

**Background:**

Studies have confirmed the rapid antidepressant action of ketamine in depressive episodes. Nevertheless, a standardized procedure for the delivery of ketamine infusion in individuals suffering from treatment-resistant depression, particularly in terms of infusion frequency and total dosage, remains undetermined. In addition, an efficacious ketamine regimen for persistent pain management involved a continuous 10-day infusion period with no notable adverse effects. Consequently, the primary objective of this study was to evaluate the antidepressant capacity of consecutive ketamine infusions spanning over three successive days, the duration of therapeutic response, and the overall safety profile of the treatment.

**Methods:**

In this randomized controlled trial, participants aged 18–64 with treatment-resistant depression were randomized to receive either intravenous ketamine or midazolam (used as an active placebo) for 40 min daily over three consecutive days. Statistical analysis using repeated measures ANOVA was employed to assess the changes in the total score of the Montgomery–Åsberg Depression Rating Scale (MADRS) and the clinical global impression-Severity from the initial assessment to 10 and 31 days post-infusion. Additionally, the duration of response and remission was evaluated using Kaplan–Meier survival analysis.

**Results:**

Out of 33 randomized participants, 20 underwent the treatment as planned. By day 10th, the ketamine group had a mean reduction in MADRS score of 12.55 (95% CI = 6.70–18.09), whereas the midazolam group had a decrease of 17.22 (95% CI = 11.09–23.36). This pattern continued to day 31, with ketamine showing a mean score decrease of 13.73 (95% CI = 7.54–19.91) and midazolam a fall of 12.44 (95% CI = 5.61–19.28). Both treatments were well tolerated, with dissociative symptoms in the ketamine group being temporary and ceasing by the end of each infusion.

**Conclusion:**

Intravenous ketamine given for three consecutive days did not show a notable antidepressant advantage when compared to the active placebo midazolam, highlighting the need for further research into effective treatments schedules for treatment-resistant depression.

**Trial registration:**

NCT05026203, ClinicalTrials.gov, registered on 24/08/2021.

**Supplementary Information:**

The online version contains supplementary material available at 10.1186/s12888-024-05951-5.

## Background

Treatment-resistant depression (TRD), as defined by the United States Food and Drug Administration and the European Medicines Agency, is the absence of a therapeutic response after two or more adequate antidepressant trials [1]. The condition affects 30–60% of individuals diagnosed with depression [2, 3]. TRD is linked to more severe outcomes than non-TRD, such as prolonged depressive episodes, increased comorbidities and substantial occupational dysfunction [4, 5]. However, the scant volume of related research hinders the identification of resistance predictors and the formulation of effective treatments [2].

Ketamine, a non-competitive *N*-methyl-D-aspartate glutamate receptor antagonist, is renowned for its ability to rapidly alleviate TRD and suicidal ideation [6]. Repetitive administration has shown a promising standardized mean difference of -0.70 (95% CI = -1.15 to -0.25) [7], and in practical settings, it has achieved response and remission rates of 45% and 30%, respectively, for depressive symptoms [8]. Ketamine is administered intravenously at a sub-anaesthetic dose of 0.5 mg/kg over 40 min, and this regimen has not been associated with major adverse events such as hypertension or bladder toxicity [9].

In 2017, the American Psychiatric Association’s Council of Research Task Force on Novel Biomarkers and Treatments endorsed a consensus for administering ketamine two or three times per week for 2 to 3 weeks for psychiatric conditions [6]. This recommendation acknowledges that the peak antidepressant effects of ketamine occur approximately 24 h post-infusion and last between 3 and 7 days [10]. However, the optimal ketamine treatment regimen remains undefined [8]. Although a Monday–Wednesday–Friday schedule is typically recommended [11], some patients and caregivers may prefer shorter treatment durations. Reflecting this preference, our clinical practice utilized both once weekly and more frequent regimens. The findings from Thailand, where the first two patients treated with once-weekly ketamine infusion for TRD showed improvement after three doses [12], align with our experience with this approach. We employed the same regimen and observed a clinical response in our patients after a similar timeframe (three doses). Notably, a 10-day consecutive daily administration of ketamine has been deemed safe for chronic pain management [13]. Leveraging previous findings on the efficacy and safety of ketamine infusion in TRD, we hypothesized that a 3-day infusion regimen could offer practical clinical benefits. This study investigated the antidepressant potency, effect durability and treatment safety of three consecutive days of ketamine infusions.

## Methods

This double-blind, randomized, active-placebo-controlled study was undertaken at Siriraj Hospital, a constituent of Mahidol University in Thailand. The institutional review board of the Faculty of Medicine, Siriraj Hospital, Mahidol University, approved the study’s protocol and informed consent form. All participants provided written informed consent before their participation. The protocol was registered on ClinicalTrials.gov (NCT05026203) submitted on 25/08/2021, and the Siriraj Research Development Fund (managed by Routine to Research: R2R) supported the study (grant number R016435052).

### Participants

Patient recruitment for this study occurred from December 2021 to August 2023 through referrals from consulting psychiatrists. The inclusion criteria were a diagnosis of major depressive disorder without psychotic features, an unsatisfactory response to two or more adequate antidepressant trials and one psychological intervention, and a minimum age of 18 years. The exclusion criteria for patients were as follows: a Montgomery–Åsberg Depression Rating Scale (MADRS) [14] score less than 24, a diagnosis of substance use disorder (excluding caffeine and tobacco) within the preceding 2 years, contraindications or allergies to ketamine or midazolam, or unstable vital signs or medical conditions.

After screening, eligible participants were randomly assigned to either the ketamine group or the active placebo group (midazolam) at a 1:1 ratio. The assignments were determined by a computer-generated randomization scheme stratified by sex and severity. Treatment assignments were concealed in sequentially numbered, sealed, opaque envelopes by the statistician NS. This approach ensured that the clinical assessors and patients were blinded to the treatment assignments.

### Intervention

To ensure patient safety and optimal treatment response, all participants underwent pre-admission COVID-19 testing and received negative results. Additionally, they were admitted to the inpatient ward for at least one night prior to their administration. This admission process helped to ensure they did not take medications that could interfere with ketamine’s antidepressant effects, including benzodiazepine, opioid antagonists, GABA-agonist, lamotrigine and propranolol. Participants were administered either ketamine or midazolam on 3 consecutive days: Tuesday, Wednesday and Thursday. They were required to fast for at least 8 h before the initial infusion. If they did not experience nausea or vomiting, fasting was not necessary before the second and third infusions. Vital signs—heart rate, blood pressure, pulse oximetry and respiratory rate—were monitored for 50 min, starting 10 min before each infusion and subsequently every 10 min. A consultant psychiatrist (K.P.) was in attendance for the entirety of each infusion session, and a study anaesthesiologist (W.M.) was available for consultation if needed. Each infusion lasted 40 min and was administered at a dose of 0.5 mg/kg for ketamine and 0.045 mg/kg for midazolam. For participants with a body mass index ≥ 30 kg/m², the ketamine and midazolam doses were calculated by adjusted body weight for participants who had BMI ≥ 30 kg/m^2^ based on sex, age and height, per established guidelines [15].

### Outcomes

Study assessments commenced on the initial assessment day (day 0). The ketamine and placebo infusions were administered on days 1, 2 and 3, enabling an appraisal of the safety and efficacy of ketamine versus the active placebo during the infusion phase. Follow-up evaluations were conducted on days 10 and 31 after the first infusion. The primary endpoint was the alteration in depression severity, gauged by the clinician-administered MADRS on day 10 after the first infusion. The secondary endpoints were the MADRS score on day 31; response rates (a ≥ 50% reduction in baseline MADRS score); remission rates (MADRS ≤ 10); and scores on the Clinical Global Impression–Severity (CGI-S) [16, 17] scales over the duration. Further secondary outcomes were assessments of vital signs and dissociative effects. The vital signs were monitored at 10-minute intervals during each infusion, followed by 15-minute intervals in the first hour post-infusion and 30-minute intervals in the subsequent hour. Dissociation was assessed with the Dissociative Experiences Measure, Oxford [18], at 20 and 40 min after each infusion.

### Statistical analysis

Drawing on prior findings on the impact of ketamine infusions given on 3 alternate days per week for up to 4 weeks [19], we projected a 15-point enhancement in MADRS scores with a standard deviation of 5.8. Setting the Type I error at 0.05 and targeting a statistical power of 0.9, we ascertained that at least nine participants would be needed in each group.

Demographic characteristics, clinical traits and baseline outcome measures were compared between intervention groups employing *t*-tests for continuous data, Mann–Whitney U tests for non-parametric data and chi-squared (𝛘^2^) tests for categorical variables. Missing data were imputed iteratively for each variable.

To assess between-group variations in MADRS and CGI scores over time, we utilized repeated measures analysis of variance. Multilevel models were applied to juxtapose group changes across all MADRS points. Intention-to-treat and per-protocol analyzes were also conducted for within- and between-group comparisons of MADRS and CGI mean scores. We employed Kaplan–Meier survival analysis to evaluate response and remission duration, defining response as a ≥ 50% decrease from baseline MADRS score and remission as a MADRS score ≤ 10. Effect sizes, calculated using Cohen’s *d*, involved dividing group mean differences by the standard deviation of baseline values for the entire cohort. The number needed to treat was also determined. All the statistical tests were two-sided, with statistical significance set at *p* < 0.05. Analyzes were executed using IBM SPSS Statistics, version 29.

## Results

### Participants

During the screening phase, 33 individuals were evaluated for eligibility, 21 of whom consented to participate (refer to the CONSORT diagram in the Supplements). After randomization, one participant was excluded from the study due to newly acquired data indicating that the individual did not meet all the inclusion criteria. The remaining 20 participants were included in the baseline assessment, and their characteristics are detailed in Table 1. Within this cohort, one participant from the ketamine group received only one of the three allocated infusions, and one from the midazolam group missed the day 31 evaluation due to worsening depression. Consequently, the intention-to-treat analysis involved 20 participants (11 in the ketamine group and 9 in the midazolam group), while the per-protocol analysis included 19 participants (10 receiving ketamine and 9 receiving midazolam). As anticipated with randomization, there were no statistically significant disparities in baseline characteristics between the groups. The ratio of participants with moderate to severe depression was 2:3, and more than half (55%) had been experiencing their current episode of depression for more than 2 years. When considering psychiatric comorbidities, four participants in the ketamine group had personality disorders, compared to one in the midazolam group. Additionally, three participants in the ketamine group and one in the midazolam group that had obsessive compulsive and related disorders.

**Table 1:** Baseline demographic and clinical profiles of patients with treatment-resistant depression: comparison between ketamine and midazolam infusion regimens

	Total (*N* = 20)		Ketamine (*n* = 11)^a^		Midazolam (*n* = 9)^a^	
	Mean	%	Mean	%	Mean	%
Female	14	70.00	7	63.63	7	77.78
Severe depression (MADRS > 34)	8	40.00	4	36.40	4	44.40
Persistent (> 2 y) in current episode ^c^	11	55.00	5	45.45	6	66.67
	Mean	SD	Mean	SD	Mean	SD
Age, y	29.35	9.18	32.36	10.62	25.67	5.61
Age at onset, y	22.80	7.67	24.73	9.11	20.44	4.95
Duration of depression, y			7.64	5.97	5.22	2.91
Education, y	17.85	2.56	18.00	3.40	17.67	1.87
Body mass index, kg/m^2^	26.40	9.45	27.74	7.88	24.75	9.30
Depression severity						
MADRS score	34.45	7.01	33.73	7.90	35.33	6.08
CGI-S score	5.05	0.76	5.09	0.83	5.00	0.71
Current number of medical comorbidities, range (0–2)	0.80	0.62	0.91	0.70	0.67	0.50
Current number of psychiatric comorbidities, range (0–3)	0.60	0.82	0.73	1.01	0.44	0.53
Current number of psychiatric medications, range [1–6]	2.85	1.18	2.91	1.04	2.78	1.39
Number of failed antidepressant trials, range [2–14]	5.40	3.20	6.18	3.89	4.44	1.88
Failed any brain stimulation trials (ECT, or rTMS, ), range (0–2)	0.45	0.69	0.64	0.81	0.22	0.44
ECT (number, %)	3, 15.00%		3, 27.27%		0, 0%	
rTMS (number, %)	4, 20.00%		2, 18.18%		2, 22.22%	
Blood pressure	Mean	SD	Mean	SD	Mean	SD
Systolic, mmHg	116.80	15.50	127.80	13.39	114.89	17.18
Diastolic, mmHg	75.00	9.70	80.82	10.94	76.00	9.22

^a^ Data are based on the intention-to-treat sample.

Abbreviations: CGI-S, Clinical Global Impression–Severity; ECT, electroconvulsive therapy; MADRS, Montgomery–Åsberg Depression Rating Scale; rTMS, repetitive transcranial magnetic stimulation.

### Primary outcome

**Fig. 1:**
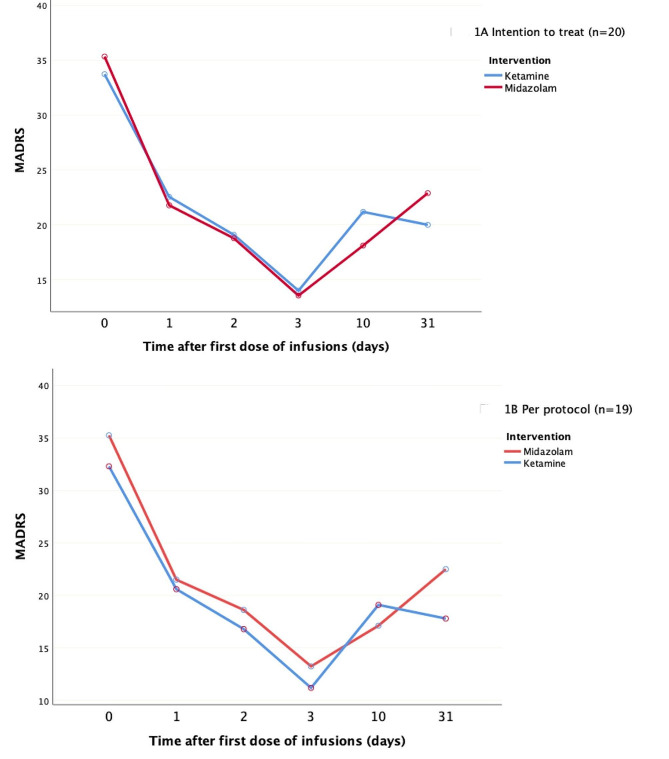
Comparative analysis of mean depression severity alterations between ketamine and midazolam treatments over a 31-day observation period

Depression scores and clinical improvements were analyzed between the ketamine and midazolam groups using intention-to-treat and per-protocol approaches (Fig. 1; Table 2). These analyzes indicated no significant differences in the reductions in the mean MADRS score between the two groups on either day 10 (3.07, *p* = 0.568) or day 31 (-2.89, *p* = 0.592). Depression severity significantly decreased across infusions in both groups (*p* ≤ 0.001; Table 3).

**Table 2:** Comparative analysis of mean Montgomery–Åsberg Depression Rating Scale (MADRS) scores between ketamine and midazolam treatments over a 31-day evaluation period

	Intention-to-treat analysis (*N* = 20)					Per-protocol analysis (*N* = 19)				
Day	Ketamine, mean (SD) (*n* = 11)	Midazolam, mean (SD) (*n* = 9)	Mean difference between groups	*p*	*d*	Ketamine, mean (SD) (*n* = 10)	Midazolam, mean (SD) (*n* = 9)	Mean difference between groups	*p*	*d*
0	33.73 (7.90)	35.33 (6.08)	-1.61	0.623		32.30 (6.67)	35.33 (6.08)	-3.03	0.317	
1	22.55 (11.66)	21.78 (8.59)	0.77	0.871	-0.08	20.60 (10.23)	21.78 (8.56)	-1.18	0.790	0.13
2	19.09 (10.96)	18.78 (7.58)	0.31	0.943	-0.03	16.80 (8.32)	18.78 (7.58)	-1.98	0.597	0.25
3	14.00 (12.00)	13.56 (5.83)	0.44	0.920	-0.05	11.20 (8.01)	13.56 (5.83)	-2.36	0.478	0.34
10	21.18 (12.98)	18.11 (9.98)	3.07	0.568	-0.27	19.10 (11.59)	18.11 (9.98)	0.99	0.845	-0.09
31	20.00 (13.86)	22.89 (8.54)	-2.89	0.592	0.25	17.80 (12.42)	22.89 (8.54)	-5.09	0.318	0.48

Abbreviations: *d*, Cohen’s *d*

**Table 3:** Intra-group variations in Montgomery–Åsberg Depression Rating Scale (MADRS) scores from baseline among patients receiving ketamine and midazolam infusions

	Intention-to-treat analysis (*N* = 20)				Per-protocol analysis (*N* = 19)			
Day	Ketamine, mean (SD) (*n* = 11)	95% CI	Midazolam, mean (SD) (*n* = 9)	95% CI	Ketamine, mean (SD) (*n* = 10)	95% CI	Midazolam, mean (SD) (*n* = 9)	95% CI
1	11.18 ** (2.15)	6.66–15.71	13.56** (2.38)	8.56–18.56	11.70** (2.29)	6.88–16.52	13.57** (2.41)	8.47–18.64
2	14.64** (2.29)	9.83–19.44	16.56** (2.53)	11.25–21.87	15.50** (2.37)	10.51–20.50	16.56** (2.50)	11.29–21.82
3	19.73** (2.52)	14.43–25.02	21.78** (2.79)	15.92–27.63	21.10** (2.49)	15.86–26.35	21.78** (2.62)	16.25–27.31
10	12.55** (2.64)	6.70-18.09	17.22** (2.92)	11.09–23.36	13.20** (2.80)	7.29–19.11	17.22** (2.95)	10.99–23.45
31	13.73** (2.94)	7.54–19.91	12.44* (3.26)	5.61–19.28	14.50** (3.12)	7.93–21.07	12.44* (3.29)	5.51–19.37

* *p* = 0.001, ** *p* < 0.001

### Secondary outcomes

Similarly, the remission rates and CGI scale results exhibited consistent patterns between and within the groups, mirroring the clinical response trends observed in the primary outcomes.

### Response and remission

**Fig. 2:**
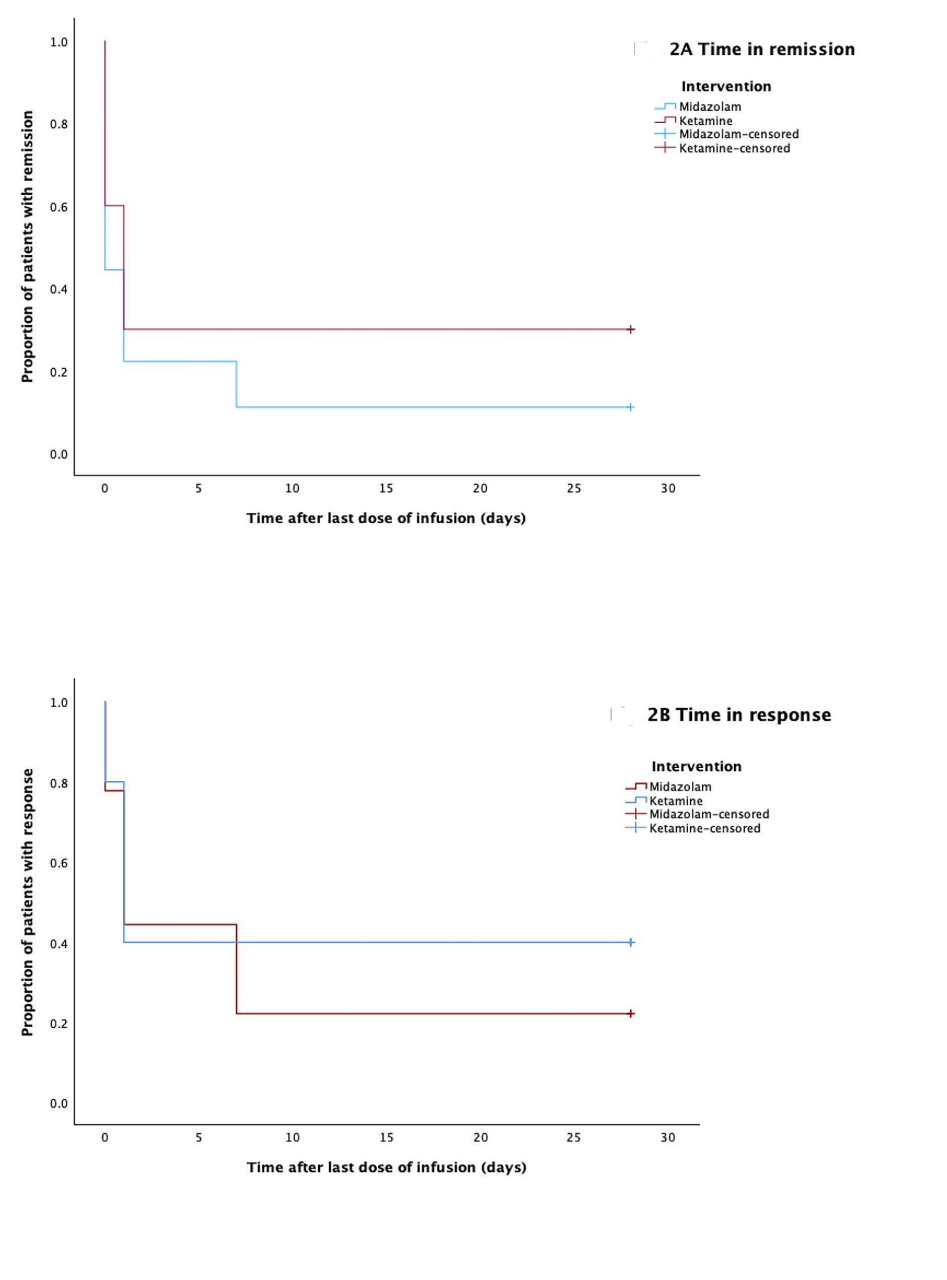
Kaplan–Meier survival estimates for (A) duration of remission and (B) duration of response in treatment-resistant depression following ketamine and midazolam therapies

Figure 2; Table 4 illustrate the durations of response and remission following treatment. Response rates, defined as a decrease in MADRS score by more than 50% from baseline, exhibited no significant variance between the groups on days 10 and 31. Despite a lack of statistically significant difference, ketamine showed a higher response rate on day 31 (36.4%) compared to midazolam (22.2%). This resulted in a number needed to treat for reponse of 7. Similarly, remission rates (participants with MADRS scores less than 10) were not significantly different between the groups on either assessment day. However, the remission rate for ketamine on day 31 (27.3%) exceeded that of midazolam (11.1%), resulting in a number needed to treat for remission of 6.2.

**Table 4:** Duration of response and remission in treatment-resistant depression following ketamine and midazolam administrations

Interventions	Mean days in remission	Lower bound	Upper bound	Mean days in response	Lower bound	Upper bound
		95% CI			95% CI	
Ketamine	8.70	0.87	16.54	11.60	3.30	19.90
Midazolam	4.11	0.00	9.80	8.11	0.97	15.26
Overall	6.53	1.49	11.56	9.95	4.36	15.53

The time-to-relapse was assessed using MADRS scores from days 1, 2, 3, 10 and 31 after the first infusion among patients who achieved a response after the last dose. Kaplan–Meier estimates for 31 days and log-rank tests revealed no statistically significant differences in relapse rates over time (𝛘^2^_1df_ = 0.312; *p* = 0.577), with mean times to relapse of 12 days for ketamine (95% CI = 3.30–19.90) and 8 days for midazolam (95% CI = 0.97–15.26). Similarly, no significant differences were found in breakthrough remission rates over time (𝛘^2^_1df_ = 0.830; *p* = 0.362), with mean durations in remission of 9 days for ketamine (95% CI = 0.87–16.54) and 4 days for midazolam (95% CI = 0.00-9.80).

### Clinical global impression-severity scale (CGI-S)

**Fig. 3:**
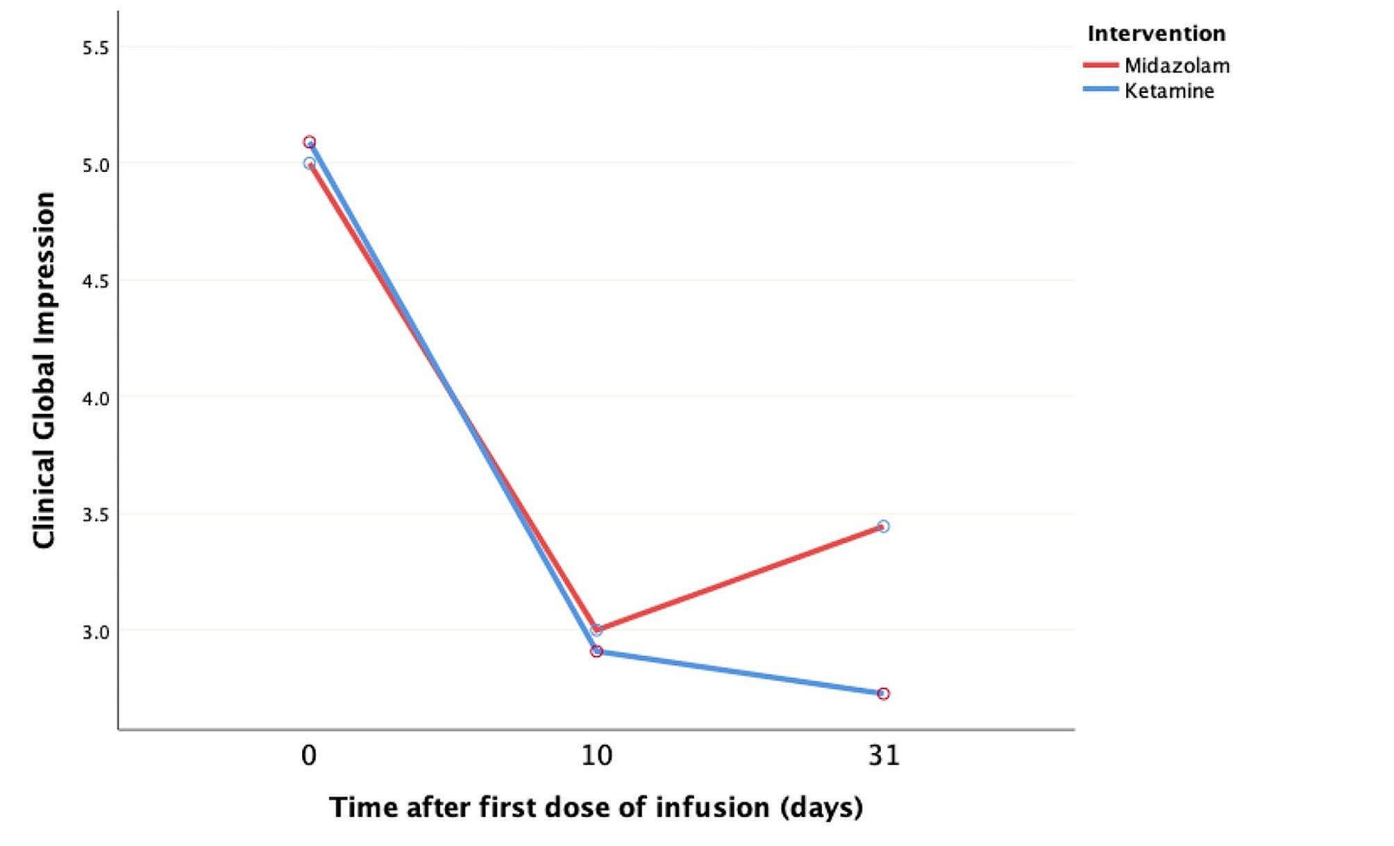
Differential mean shifts in Clinical Global Impression-severity (CGI-S) scores following ketamine versus midazolam administration across a 31-day interval

CGI-S scores demonstrated significant improvements from baseline to the end of the study in both the ketamine and midazolam groups (*p* ≤ 0.01; Fig. 3). Despite these improvements, there was no statistically significant difference in the change in CGI-S score between the two treatment groups. Specifically, the ketamine group experienced a reduction in CGI-S scores from 2.60 on day 10 to 2.40 on day 31, indicating improvement. Conversely, in the midazolam group, the CGI-S score increased from 3.00 to 3.44, indicating worsening.

### Adverse events

The infusion phase of ketamine was associated with several common side effects: general malaise (72.7%), emotional overwhelming (18.2%), increased blood pressure (18.2%), and nausea or vomiting (9.1%). Despite reporting no thoughts during the infusion, two participants who experienced overwhelming emotions described feeling sudden and extreme sorrow during the infusions. Watching an interesting and relaxing video beforehand appeared to prevent these overwhelming emotions. One participant (9.1%) with morbid obesity experienced oxygen desaturation during the three-day ketamine infusion regimen. Participant’s oxygen saturation levels decreased by 3–6% from baseline each day. Simple interventions, including gently arousing and postural adjustment, helped return the oxygen saturation levels to normal. None of these adverse events were life-threatening. For those suffering from nausea, intravenous ondansetron was effectively used to relieve the symptoms. In the midazolam group, somnolence was reported by all participants (100%) during the infusion phase.

### Vital signs

Ketamine administration was associated with a temporary rise in systolic and diastolic blood pressures (SBP and DBP). After ketamine administration, the mean peak SBP reached 144.18 mmHg (SD = 2.78), while the mean peak DBP was 88.45 mmHg (SD = 12.56). In contrast, midazolam administration resulted in lower mean peaks, with an SBP of 113.78 mmHg (SD = 10.57) and a DBP of 72.78 mmHg (SD = 9.11). The ketamine group experienced a statistically significant increase in the mean SBP (16.36 mmHg; SD = 14.59, *p* = 0.004) from baseline. However, the increase in the mean DBP in the ketamine group was non-significant, with a mean difference of 7.64 mmHg (SD = 15.16, *p* = 0.104). Conversely, the midazolam group showed a transient decrease in blood pressure, with the largest mean decrease in SBP being 1.11 mmHg (SD = 9.32, *p* = 0.730) and in DBP being 3.2 mmHg (SD = 5.70, *p* = 0.128).

One participant with a BMI greater than 30 kg/m² in the ketamine group exhibited a blood pressure increase exceeding 30% from baseline, which was resolved by reducing the infusion rate. No blood pressure elevations required antihypertensive treatment. Heart rate differences between the groups were not statistically significant (data not shown).

### Dissociative experience

During the three infusions, slightly greater dissociation scores were observed in the ketamine group than in the midazolam group at 20 and 40 min, as measured by the Dissociative Experiences Measure, Oxford. However, these increases were not statistically significant, and the differences in scores resolved within 10 min after the end of each infusion (data not shown). There was a participant in the ketamine group experienced vivid visual hallucination, which resolved immediately after the infusion concluded. The study did not report any post-infusion delusions, hallucinations, or manic episodes.

## Discussion

This study revealed unexpected results regarding the efficacy of ketamine in mitigating depressive symptoms in comparison with the active placebo, midazolam. Both interventions successfully reduced depressive symptoms, with no significant difference evidenced by MADRS or CGI-S scores. However, the response rate in this investigation, 36.4%, contrasting with that of a prior study utilizing a six-dose regimen of ketamine [20]. Additionally, the remission rate attained in our investigation aligned with the documented real-world effectiveness of ketamine (28.9%) [8].

A conceivable explanation for the divergent outcomes observed in our study compared to previous research might be attributed to the lower number of ketamine doses administered. We administered daily ketamine infusions over three consecutive days and noted a 36.4% response rate 4 weeks after the third infusion. This rate falls short of the 80% response rate associated with 4 infusions documented in an earlier study [21]. In that study, half of the responders achieved a response with only one or two infusions, but the remainder needed all 4 infusions. Hence, to optimize the response within 4 weeks, a minimum of 4 ketamine infusions seems essential. An alternative hypothesis explaining the insignificant results between groups is that repeated ketamine infusion for three consecutive days may lead to ketamine tolerance. Although several studies [20, 22] have demonstrated that repeated ketamine administrations do not show evidence of tolerance, their regimens were less frequent compared to ours, such as 1–2 times weekly. Frequent use of ketamine can result in upregulation of ketamine-metabolizing enzymes and neuronal adaptation, resulting in a diminished response and a requirement for higher ketamine doses [23, 24]. Tolerance to ketamine in a patient with depression has also been reported [25]. Conversely, administering infusions over three consecutive days might yield enhanced antidepressant effects with an increased dosage, such as 0.75 mg/kg, as suggested by prior research [26, 27]. The so-called ‘contextual effects’ [28], potentially influenced by the treatment environment, may account for the improvements observed in our study groups, both immediately and in the short term. By day 31, there was an increase in MADRS scores among participants in our midazolam group, in contrast to the ketamine group, which experienced only minimal changes in scores.

Several other factors might have influenced our findings especially the level of treatment refractoriness [29]. Specifically, the ketamine group had greater chronicity of depression, a higher number of unsuccessful medication trials, and more participants who failed electroconvulsive therapy treatment (3 vs. 0) compared to the midazolam group. Furthermore, evidence suggests that younger patients may achieve more favorable therapeutic outcomes than older individuals [8]. In our study, the ketamine group had a higher mean age than the midazolam group, which might have influenced the therapeutic effects observed. However, these factors were not statistically different between two groups, and a larger sample size might be needed for clearer conclusions. Given the ongoing COVID-19 pandemic throughout the study period, the stress experienced by participants could be a confounder affecting the results. This unprecedented stressor has impacted people in various ways, including through social isolation and economic crisis that arose during the pandemic. Furthermore, the use of midazolam as an active placebo may result in a relatively smaller average antidepressant effect compared to ketamine. While midazolam possesses recognized anxiolytic properties, its potential antidepressant effect remains unclear. Although some evidence suggests it might be more effective than normal saline [30], further research is needed to understand its role in depression treatment. Finally, a longer interval between each ketamine infusion may be necessary to allow sufficient time for downstream molecular and intracellular processes to promote neuroplasticity, which is pivotal in clinical improvement [31]. Our findings suggest that for optimal efficacy in treating TRD, the interval between ketamine infusions should ideally exceed 24 h.

While this pilot study aimed to assess the feasibility and gather preliminary data on the effectiveness of three consecutive days of daily ketamine infusions for TRD, there were some limitations to consider. The sample size was designed for moderate statistical power, suitable for a pilot study, but might be underpowered to assess treatment efficacy. The reluctance of severely depressed patients to participate in a randomized, placebo-controlled trial reduced our ability to discern definitive differences between treatments and associated response factors. Additionally, only 18 of the 33 recruited patients (54.5%) completed the study, largely due to the impact of the COVID-19 pandemic and subsequent isolation measures [32, 33]. Worth noting is that the diversity in antidepressant regimens among participants could have confounded the results, though this was mitigated by stabilizing medications for at least four weeks before starting the infusions. Finally, the absence of a rating scale for depression subtypes limited our understanding of the influence of depression subtypes on treatment outcomes. This approach was adopted due to the constraints imposed by the limited sample size of the study.

Despite its limitations, our study possesses notable strengths. This is the first study to investigate a daily ketamine infusion protocol over 3 consecutive days, revealing no superior antidepressant effects compared to an active placebo. Employing a double-blind, active placebo-controlled design was crucial for evaluating this new treatment protocol before its potential clinical application. In addition to explore a new ketamine treatment frequency, this study stands as the first RCT of ketamine performed in Thailand. This broader perspective on ketamine treatment is valuable. While a growing body of research explores intravenous ketamine for TRD, most studies are limited to Western or high-income countries [34, 35]. By including participants from Thailand, this study contributes to a more comprehensive understanding of treatment effectiveness and potential adverse events across diverse backgrounds. Furthermore, publishing non-significant results is vital because it contributes to the scientific literature and informs clinical decision-making. Although the findings were non-significant, they provide essential safety data for ketamine administration over three consecutive days in this population. Finally, this research was initiated due to patient and family concerns about the need for a feasible dosing regimen. By addressing these issues, the study offers valuable insights that could enhance treatment experiences for individuals with TRD.

## Conclusion

This pilot study successfully assessed the feasibility and tolerability of a three-day daily ketamine infusion protocol for treatment-resistant depression. While the results provided preliminary data on its potential effectiveness, the limited sample size precludes definitive conclusions about its comparability to existing treatments. Future research should explore two key areas: expanding the sample size and investigating the potential of ketamine regimens with different dosing frequencies and quantities for alleviating depressive symptoms.

### Electronic supplementary material

Below is the link to the electronic supplementary material.

Supplementary Material 1

## Data Availability

Data is provided within the manuscript or supplementary information files.

## Abbreviations

BMI: body mass index
CGI-S: Clinical Global Impression–Severity
DBP: diastolic blood pressure
MADRS: Montgomery–Åsberg Depression Rating Scale
SBP: systolic blood pressures
SD: standard deviation
TRD: treatment-resistant depression
