# Supplementary material for: A randomized controlled pilot study of daily intravenous ketamine over three days for treatment-resistant depression
Source: BMC Psychiatry. 2024 Jul 18;24:512. doi: 10.1186/s12888-024-05951-5 (PMC11256507; doi:10.1186/s12888-024-05951-5)
Supplement: Supplementary file 1 — Supplementary Material 1 [file 12888_2024_5951_MOESM1_ESM.docx]

CONSORT diagram for the trial. ITT = intention-to-treat group; PP = per-protocol group.

Assessed for eligibility

(*N*=33)

# Enrolment

Excluded (*n*=12)

- Did not meet inclusion criteria (*n*=8)
- Declined to participate (*n*=4)

Randomised (*n*=21)

#

Day 10 (*n*=9)

Day 31 (*n*=8)

- Lost to follow-up due to worsening of symptoms

Analysed ITT (*n*=9)

Analysed PP (*n*=9)

Analysed ITT (*n*=11)

Analysed PP (*n*=10)

Day 10 (*n*=10)
Day 31 (*n*=10)

Allocated to ketamine

(*n*=11)

- Received the allocated intervention (*n*=10)
- Received only one infusion (*n*=1)

Excluded (*n*=1)

- Identified a history of bipolar disorder while receiving midazolam

Allocated to midazolam

(*n*=10)

- Received the allocated intervention (*n*=9)

**Follow-up**

# Analysis

Allocation
